# Client experiences of a task-shifting supported self-management intervention for depression in Vietnam

**DOI:** 10.1186/s12913-024-12036-2

**Published:** 2024-12-18

**Authors:** Leena W. Chau, Hayami Lou, Jill K. Murphy, Vu Cong Nguyen, Will Small, Hasina Samji, John O’Neil

**Affiliations:** 1 Faculty of Health Sciences, Simon Fraser University, Vancouver, Canada; 2 Interdisciplinary Health Program, St. Francis Xavier University, Antigonish, Canada; 3 Institute of Population, Health and Development, Hanoi, Vietnam

**Keywords:** Task-shifting, Task-sharing, Depression, Client experiences, Lay health worker, Vietnam

## Abstract

**Background:**

The global burden of mental illness is substantial, with depression impacting close to 300 million people worldwide. This has been exacerbated within the context of the COVID-19 pandemic. Yet, in many low- and middle-income countries including Vietnam, there is a substantial treatment gap, with many requiring mental health care unable to access it. Task-shifting is an evidence-based approach that seeks to address this gap by utilizing non-specialist providers to provide care. While there is a large body of literature exploring task-shifting, there is little that explores the client experience. This paper describes the facilitators and barriers impacting the client experience of a task-shifting supported self-management (SSM) intervention for depression in Vietnam. SSM involves a client workbook and supportive coaching by non-specialist providers.

**Methods:**

This paper is situated within a randomized controlled trial that demonstrated the effectiveness of the SSM intervention in adult populations across eight provinces in Vietnam. Semi-structured interviews were conducted with a convenience sample of clients (recipients of the intervention) with depression caseness as measured by the Self-Report Questionnaire-20 depression screening measure, and providers (non-specialist “social collaborators”) to explore SSM’s acceptability and factors influencing participation and adherence. This paper presents the qualitative findings from an analysis of the interviews, focusing on the client perspective. Qualitative descriptive methods and thematic analysis were used.

**Results:**

Forty-five clients were interviewed. Sub-themes reported for the facilitators and benefits for the client experience of the SSM intervention were *client-provider relationship building* and *family and community connections*. Sub-themes reported for the barriers were *clients’ responsibilities*, *clients’ health conditions*, and *consequences of stigma*.

**Conclusions:**

Due to challenges with sustaining and scaling up the in-person SSM intervention in Vietnam, the research team has pivoted to delivering the SSM intervention digitally through a smartphone-based app adapted from SSM, with direction from the Government of Vietnam. Findings from this study suggest that while digital interventions may support accessibility and convenience, they may neglect the critical human contact component of mental health care. Ultimately, a model that combines digital delivery with some form of human contact by a support person may be important.

**Supplementary Information:**

The online version contains supplementary material available at 10.1186/s12913-024-12036-2.

## Background

### Introduction

The global burden of mental illness is substantial, accounting for 32.4% of years lived with disability and 13.0% of disability-adjusted life-years, according to Vigo et al., [1]. There are various social and economic factors that contribute to the burden of mental health conditions, including poverty, gender-based discrimination, urbanization, internal migration, and lifestyle changes [2]. Depression, the most common mental health condition, affects over 300 million people globally and is a major contributor to the global burden of disease [3]. In Vietnam, a low- and middle-income country (LMIC), the limited available epidemiological evidence suggests that depression prevalence is comparable with global rates [4]. The COVID-19 pandemic also contributed to a considerable increase in the burden of depression worldwide [5]. Depression increases the risk of suicide and is the top contributor to disability globally [6]. The World Health Organization (WHO) launched the Mental Health Action Plan 2013–2030 in response to the Sustainable Development Goals, with the aim of promoting mental health and well-being for people of all ages [6].

In many LMICs, including Vietnam, there is a substantial gap in available and accessible care for people experiencing mental health conditions [7, 8]. LMICs often lack the resources to effectively implement mental health policies and programs, making access to mental health care difficult for citizens [9]. Those with mental health conditions in LMICs often face discrimination and may be stigmatized due to low mental health awareness and mistreated or abused, negatively affecting their willingness to seek appropriate care and adhere to treatments [10]. With 92 million inhabitants, Vietnam is the 14th most populous country globally [11]. Vietnam has seen significant progress in improving the economic and social well-being of its people, with the World Bank praising its “spectacular success” in poverty reduction and economic growth over the past 15 years [1]. However, as the government prioritized infrastructure projects most directly connected to economic development during the country’s shift to an industrial economy, social services and healthcare – including mental health –received limited investment [6, 12]. Despite overall economic growth, access to healthcare has become increasingly difficult as the population grows and demand for services increases, with some parts of the system being privatized and receiving fewer government subsidies [13]. Similar to other LMICs, Vietnam is experiencing a significant shortage in mental health professionals; a study examining the prevalence of psychiatrists, nurses and psychosocial care providers (which includes psychologists and social workers) in 144 LMICs showed that Vietnam had the largest shortage of psychiatrists and psychosocial care providers, with 1.70 psychiatrists and 11.52 psychosocial care providers per 100,000 of the population [14].

Task-shifting or sharing is an evidence-based approach that helps to address the gap in health human resources. Task-shifting interventions for mental health are defined as those being provided by non-specialist providers who have received training specific to the interventions being delivered [15]. Currently, there is a large body of literature detailing their roles in international settings, with several interchangeable terms being used for these ‘lay health workers’ [16–18]. This includes the ‘social collaborator’ role, which is unique to Vietnam, and their capacity in the delivery of mental health services [19, 11]. Importantly, while there is a large body of literature on task shifting and the role and motivations of the providers [17, 20, 21], there is little that explores the client experience. Understanding the client experience is increasingly recognized as being crucial to delivering effective and high-quality healthcare [22, 23].

This paper will describe the contextual facilitators and barriers that impact the client experience of a task-shifting supported self-management (SSM) intervention for depression (described below in “Prior Work”) in Vietnam, through semi-structured interviews with clients and providers of the intervention. Contextual facilitators and barriers will elucidate evidence on the SSM model’s sustainability and factors impacting its scale-up in the Vietnamese cultural and social context. Findings support enhancement of client-centred mental health care delivered using task-shifting methods in community-based settings in Vietnam, along with other similar settings [24].

### Prior work

This paper is situated within ongoing work evaluating the effectiveness and scaling-up of a Supported Self-Management (SSM) intervention for adult depression in community-based settings in Vietnam. SSM is defined as “access to a self-management guide (workbook or website) plus encouragement and coaching by a health care provider, family member, or other supporter” [24]. SSM is based on principles of cognitive behavioural therapy and utilizes task-shifting approaches. The *Mental Health in Adults and Children – Frugal Innovations* (MAC-FI) study, funded by Grand Challenges Canada (GCC - April 2016-March 2019) demonstrated the effectiveness of the SSM intervention in adult populations in Vietnam through a randomized controlled trial (RCT) across 8 provinces [25]. The RCT used a cluster-randomized modified stepped wedge design and included 376 adults in 32 communes (4 in each province) across the 8 provinces. Provinces (3 northern, 3 central, and 2 southern) and communes were selected in collaboration with MOLISA for geographic, cultural, and socioeconomic diversity. Local implementation was led by the Institute of Population, Health and Development (PHAD), a leading non-governmental research organization in Vietnam and collaborator on the project.

In the MAC-FI SSM intervention, client participants were provided an “Antidepressant Skills Workbook” (ASW) that utilizes three key principles of: (1) reactivating your life; (2) thinking realistically; and (3) solving problems effectively, along with supportive coaching by local community-based social collaborators and social workers from the Ministry of Labour, Invalids and Social Affairs (MOLISA). Social workers usually have a four-year Bachelor of Social Work degree, while there is substantial variation in the training and experiences of social collaborators. Social workers have a wide range of administrative responsibilities and thus have limited time for direct service provision. Due to this, social collaborators were mainly responsible for providing individual coaching on the use of the ASW in the clients’ homes, over the intervention’s two-month period. Social collaborators were selected in consultation with MOLISA. Social collaborators were usually highly respected, often retired community members with backrounds in sevice provision or roles with community organizations, who received 3 days of training provided by the study team and a psychiatrist from a psychiatric hospital, along with supervision and support from the social workers. Training involved screening for depression using the SRQ-20 and delivery of the coaching intervention including principles of the ASW.

The number of social collaborators involved varied depending on the size of the commune (a municipal subdivision), with six to ten collaborators per commune. The coaching sessions with clients were scheduled every two weeks, during which the social collaborator discussed the client’s progress, reviewed the concepts covered in the ASW, and assisted in creating a plan for the following two weeks. At 2 months, 26.4% of the intervention group and 42.3% of the delayed group (control) had SRQ-20 scores > 7. SRQ-20 is a 20-item instrument developed by WHO to screen for psychological disturbance, including for depression [26]. The adjusted odds ratio of having depression between the intervention and control was 0.42 (*p* < 0.0001), 95% CI (0.28, 0.63). Receiving the intervention thus reduces the odds of having depression by 58% after 2 months [25]. As a follow-up to MAC-FI, the *Implementation Research to Improve Scale-Up of Depression Services in Vietnam (IRIS-DSV)* project (April 2019-in progress), is a five-year, qualitative implementation science study funded by the Canadian Institutes of Health Research. The purpose of the IRIS-DSV project was to interview participants from the MAC-FI RCT to understand factors influencing the potential scale-up of the intervention beyond the MAC-FI trial.

## Methods

### Setting

This paper is based on a qualitative analysis of semi-structured interviews conducted from November 2018 to May 2019 in Vietnam for the IRIS-DSV project. In the IRIS-project, semi-structured interviews were conducted with a convenience sample of clients and providers of the SSM intervention tested in the MAC-FI RCT.

### Sample

A convenience sample of 92 key-informant interviews with 45 clients and 47 providers regarding their experiences with the SSM were completed. While we originally attempted to conduct purposive sampling for representative distribution based on characteristics such as sex and rural versus urban residency, we encountered two issues: (1) most of the participants recruited into the MAC-FI RCT were women and (2) given the time elapsed between the MAC-FI RCT and the IRIS-DSV qualitative study and consequently reduced interested participant pool to recruit from for IRIS-DSV, we had to pivot to convenience sampling.

Of the 45 clients interviewed, 91.1% were women and 37.5% were over 55 years of age. This is comparable with the full client sample in the MAC-FI RCT (*n* = 376), where 84.5% were women and 18.7% were over 55 years of age. Inclusion criteria included: (1) having been screened positive for depression (scoring ≥ 7 on the SRQ-20) and (2) subsequently referred to the SSM intervention in the MAC-FI RCT. Exclusion criteria included: (1) cognitive impairment; (2) symptoms of severe depression or suicidal ideation or psychosis; (3) impaired vision; and (4) illiteracy.

Interviews were also conducted with a convenience sample of 47 providers from among the 393 social workers and social collaborators who were trained to deliver the SSM intervention as part of the MAC-FI RCT. Of the providers interviewed, 78.7% were women and 48.9% were over 55 years of age. This is comparable to the full sample in the MAC-FI RCT, where 81.5% were women and 46.6% were over 55 years of age. Inclusion criteria included being trained to deliver the SSM intervention and having delivered it to three or more clients to ensure depth of experience with the intervention.

### Instruments and procedures

Interviews with clients (*n* = 45) focused on the acceptability of the intervention, the relationship with the provider and the usability of the materials, along with factors influencing participation and adherence. See Appendix A for the Interview Schedule for Clients. A member of the PHAD research team in Vietnam invited clients to participate in qualitative interviews using the Client Recruitment Script by either telephone or email, based on client preference. See Appendix C for the Client Recruitment Script. Recruitment was undertaken through commune health staff, who generated a list of possible clients who were involved in the MAC-FI project, from which participants were selected using convenience sampling. If a client declined to participate, another client was selected using the same method. All clients were asked questions regarding their experiences with the screening and referral processes. If the client completed the intervention, they were asked about their experiences using the SSM materials, their relationship with the SSM provider, and perceived benefits of participating in the intervention. For clients who did not complete the intervention, they were asked their reasons for non-adherence and cessation.

Interviews with the providers (*n* = 47) included an examination of the roles and functions of social collaborators, which have been published elsewhere [11]. However, these interviews were also coded for commentary on the client experience and these data are included in this paper. See Appendix B for the Interview Schedule for Providers. Similar to recruitment of clients, a member of the PHAD research team in Vietnam invited providers to participate using the Provider Recruitment script by either telephone or email, depending on their preference. See Appendix D for the Provider Recruitment Script.

In most cases, clients were willing to share with interviewers when asked about the situations surrounding and contributing to their mental state. In that vein, interviewers were very consistent in asking clients about their current situation and how they were feeling, prompting about specific client circumstances if the interviewer was familiar with the participant. Interviewers also asked questions about client overall wellbeing, self-reported mental health, and family conditions. They often provided suggestions and advice to the clients during the interview, such as how to keep up their progress and how further participation in community events would be helpful. Interviews were conducted following the Client Interview Schedule, and were enhanced through the field notes captured by the interviewers, which helped gather rich details about clients’ personal lives and circumstances. This supported the development of the client profiles as detailed below in the Results.

All interviews were conducted by Vietnamese staff from the Institute of Population, Health and Development (PHAD) in Vietnamese, after obtaining informed consent from the participants. All interviews were recorded, with approval from the participants, and were transcribed verbatim and translated into English by either a translation service or PHAD team members who did not take part in the interview. However, the quality of translation was variable, and transcripts have been further corrected by native English-speaking members of the research team to ensure legibility. All corrections were reviewed and approved by a Vietnamese speaking staff from PHAD to ensure accuracy and cultural relevancy.

### Ethical approval

In Vancouver, Canada, all procedures were approved by the SFU Research Ethics Board [2015s0604 and 2018s0340] and the Harmonized Research Ethics Board BC [H20-02658]. In Hanoi, Vietnam, all procedures were approved by the PHAD Institutional Research Ethics Board [2016/PHAD/MAC-FI-AD-01 and 2019/PHAD/IRIS-01].

### Data analysis

Qualitative descriptive methods and thematic analysis [27] were used to examine the key informant interviews. All interview transcripts were entered into NVivo for coding [28]. A coding framework outlining broad themes corresponding to our theoretical framework and the findings of the MAC-FI study and the literature were developed deductively. Inductive analysis was undertaken to identify sub-themes and the specific characteristics of barriers and facilitators. Two team members (HL and LC) independently read, re-read, and coded the translated interview transcripts. Key codes were identified and captured in a coding guide. Through an iterative process involving numerous rounds of discussion and consultation amongst the team members, the codes were identified, categorized, and collapsed into larger themes, which were then discussed and agreed upon by the team. To enhance rigor and trustworthiness, an interrater reliability test was performed, and the coding process was documented in detail.

## Results

### Client demographics

Of the 45 total clients interviewed, 4 clients identified as male (8.9%) and 41 identified as female (91.1%). This is fairly representative of the gender composition of the sample from the MAC-FI RCT, where 15.4% identified as male [29]. Clients ranged between 24 to 67 years of age. One client did not report age. Occupations reported included: farmer, teacher, housewife, construction worker, freelance work, amongst others.

### Client profiles

Several client profiles are provided here as examples to illustrate some of the circumstances and difficulties they experienced while dealing with depression. Given the stigma surrounding mental health issues, most clients were unwilling to disclose personal details of their lives, so profiles were selected from a relatively small cluster of the client sample where the interviewer was able to elicit a discussion of personal circumstances. Clients were assigned a letter of the alphabet to protect their confidentiality. Clients are described by these, along with their province.

#### Client A (Female [F], Ben Tre)

Client A has a husband who is illiterate and an adult daughter who is currently not working. She finds it difficult to focus and feels like “she wants to die in crowded places”. She used to be able to go to the market but now feels it is too overwhelming. She joined the project to get better, but her husband discouraged her from participating.

#### Client B (F, Ben Tre)

Client B moved to her current village when she got married at the age of 18. Client B is a caretaker for her husband who is ill and requires the use of a wheelchair. She struggles to balance this with her job at the market selling soy milk. Before attending the interview, she finished selling her wares for the day and dropped off her husband at the hospital, as he had a stroke 10 days prior. She previously had support and help from her daughter, but since her daughter recently got married, all of the responsibilities have fallen to Client B. Although she reported she did not remember many aspects of the intervention and that it was difficult to find time to read the workbook due to the busy nature of her life, she reported feeling satisfied with the experience and has recommended it to other people in her life experiencing depression.

#### Client C (F, Thanh Hoa)

Client C is a 57-year-old farmer with three teenage children. Her husband works in another province to support the family financially, while Client C looks after their children and the family farm and sells scrap iron. She finds herself worrying about the future and reported often only sleeping around two hours at night. She mentioned her children are busy with their studies, so she does not ask them for help when reading the workbook, although she would have appreciated their assistance. After taking part in the project, she gained a better understanding of depression as an illness, and reported that both her quality of sleep, as well as her relationship with her husband, had improved.

#### Client D (F, Thanh Hoa)

Client D is a recent widow and the breadwinner of her extended family, which includes her children and elderly cousins. She and her family often face financial struggles, which leads her to continue to work even in her own old age. Despite this, she is proud that her children are able to rely on her. Client D has been living with feelings of stress and despair along with heart problems for an extended period of time. She mentioned that she began participating in the project soon after her husband’s death, who was her strongest support and who she often shared her struggles with. Client D reported she was satisfied with the care by the social collaborators and the content of the intervention, including how the workbook taught her about managing her health. However, she also shared she had difficulty finding hope for a better future due to her increasingly poor health and worsening financial situation, with the lack of support she receives from the government being a significant contributor to her struggles.

#### Client E (F, Quang Ninh)

48-year-old Client E lost her husband suddenly four years ago to cancer, with her mother-in-law passing away soon after. Client E’s son was only 2 years old at the time of her husband’s passing, when she was suddenly put in the position of needing to become a breadwinner to her young son, ill mother-in-law, and her stepchildren, who began to treat her poorly after their father’s death. Client E still mourns her husband, sharing that she often holds her son and cries. After her losses and being left alone with young dependents, she fell into a deep depression and did not feel as if she could continue on. She then received support from the social collaborators, who reached out to her and continued regular visits to her home following the conclusion of the SSM intervention period. The social collaborators encouraged her to join her local women’s union, where she received the social support she had been severely lacking. At the time of the interview, she had moved in with her parents, her child was enrolled in kindergarten, and she relied on seasonal work to make ends meet.

#### Client experiences of the SSM intervention influencing participation and adherence

Clients and providers identified two key facilitators and benefits influencing clients’ participation and engagement with the SSM intervention: 1) client-provider relationship building and 2) family and community connections. Participants also identified four barriers to clients’ participation and engagement: 1) clients’ responsibilities; 2) clients’ health conditions; and 3) consequences of stigma. See Table 1 for a summary. These sub-themes are described below within the main themes *Facilitators and Benefits,* and *Barriers*.

**Table 1:** Summary of themes and sub-themes

**Facilitators and Benefits**	
Client-provider relationship building	
Family and Community Connections	
**Barriers**	
Clients’ responsibilities	
Clients’ health conditions	
Consequences of stigma	

### Theme: facilitators and benefits

#### Client-provider relationship building

A key sub-theme supporting engagement with the SSM intervention was the client-provider relationship. Clients largely reported they were able to build social rapport with their providers. Most of the feedback through client interviews was focused on the providers, rather than the intervention itself. A strong provider was identified by clients as someone who takes the time to get to know them, their families, as well as their living conditions and life circumstances. Due to the nature of the client-provider relationship and the fact that individuals with depression can be hesitant to socialize, it is up to the providers to take the lead in establishing a positive initial interaction and a relationship through friendliness and openness. Clients reported positively on providers who demonstrated enthusiasm, motivation, and strong interpersonal skills. Another important quality reported by Clients is persistence, especially through initial rejections by clients. As Client J reported:

“At first, I was bothered [and] did not want to talk… I wanted to avoid [the provider], but seeing her [visit] many times, she was patient. If she [visits] in the early morning but [I am unavailable], she will come late at night. After [seeing] that, I began to talk with her.” (Client J, Quang Nam)

One key factor that outlined successful client-provider relationships was the importance of providers building trust and rapport with clients over time.

“I have to dedicate [time] and be subtle when visiting a client… [especially] if you meet their family. You could make some [jokes] or have lunch with them. And then when their family [goes] out, [leaving] you and [the client], you could start to talk to them. They will be more open and won’t be embarrassed.” (Provider F, Quang Nam)

#### Family and community connections

Another sub-theme supporting engagement with SSM was the importance of family and community connections. Clients identified strong social collaborators as being members of the communities in which they live and work. Because social collaborators live in the community and often hold concurrent jobs as commune health workers and/or community representatives, they have existing relationships with the clients. They may have a history of previously working with them on other local health initiatives and may be on friendly terms, or at least familiar with the client and their life circumstances. Providers working in their home communities have the benefit of existing connections, so they are not building trust from the ground up.

“Q: [Did] you already know these [social] collaborators?

A: [Yes, because we are] from the same village and hamlet, we know each other.”

(Client K, Khanh Hoa)

“Q: The first time you met them, did they come to you or [the] clinic?

A: I came to their house, not only because I’m the [social] collaborator, but also because I’m the head of the village, so I understand and know all the [family] situations in the village. So [if anyone] needs to talk to [me], I will [go] to their house to meet them and talk.” (Provider G, Da Nang)

Beyond introducing and guiding the client through the SSM intervention, social collaborators often spend their home visits conversing and getting to know the client. Several providers discussed the importance of approaching a client with sensitivity so as to avoid embarrassment and rejection from the client’s side.

“Q: When you approached the client at first, did you find any difficulties and advantages?

A: There are some difficulties. Although I [visited] many times, they are afraid, do not talk [to me or] speak directly on their problem. I also try to ask [about] their health and family. I support them but not directly speak to the problem [of depression]. If [my initial] approach is not done correctly, they will resist, and it will be difficult for me to work with them.” (Provider H, Ben Tre)

“Q: How do you improve the [client-provider] relationship?

A: Usually, I make acquaintance with them, and [share] a little bit about myself. Then I [discuss] social matters I read in the newspapers. Gradually they will [become] friendly with me and tell their stories. Sometimes they talk and cry. I [give] advice and then find [solutions], inviting them to join in the women’s union meetings… they [become] friendly with me.” (Provider I, Ben Tre)

Providers encouraged clients to engage with groups and activities in the community such as the local Women’s Union. In addition to guiding clients to build local connections, providers also spent time with clients engaging in social activities outside of official SSM related visits.

“The [SSM] program gave us a chance for us to gather and discuss. [The providers] visit and talk with us, showing the ways to relax. It is so fun. She came to me and gave me advice every time I cried. She helped me all the time, [even when] I thought it was too hard for me to bring up my children and also have to handle negative thinking. I [feel] better because of them.” (Client L, Da Nang)

As other clients reported, “Before, I did not do anything, [only] staying at home whenever I was sick. But I joined the Inter-Generation Club, so [now] I go to gymnastics every afternoon and then cook in the evening,” (Client N, Thanh Hoa) and “It is more fun to join [local] activities. Because in the past, I worked [providing] social activities for the Youth [and] Women's Unions. When I fell down, everyone encouraged me to stand up.” (Client M, Quang Ninh)

### Theme: barriers

In addition to the facilitators and benefits influencing participation and adherence mentioned above, participants also spoke to some of the contextual barriers, described below.

#### Clients’ responsibilities

One of the main challenges that providers identified was the difficulty in finding the right time to engage with clients. Providers and clients themselves expressed that many clients were busy with work, supporting their families, and meeting basic needs (of which failure to do so was often identified as contributing to their depression).

“Q: Do you remember any skills [from the workbook]?

A: It has been a long time, and I’m also too busy with my job at the market.

Q: You have to look [after] your husband, and sell soy milk at the same time?

A: Yes, I have to do it all by myself. My daughter got married.” (Client O, Ben Tre)

This posed a challenge to finding appropriate times for providers to visit the clients, with providers reporting struggles working around client schedules. Sometimes providers had to visit a client multiple times before the client was available to engage with them, and providers were not compensated for the additional time and transportation costs. One provider reported, “Based on the client their schedule, also their work [hours] and mine, I have to make it work for both of us.[…] I have to choose a time that would work for both of us.” (Provider S, Da Nang)

#### Clients’ health conditions

Many clients shared how they live with concurrent medical conditions, often physical, in addition to suffering from depression. These conditions ranged from those that caused significant long-term pain and mobility issues, to conditions that could be remediated but were left untreated, such as poor eyesight leading to clients being unable to read the workbook. Often this left clients dependent on help from providers and their family members in order to participate in the intervention. For example, one client spoke to issues with their leg, “Yes, I did wake up early and do some exercise, but then my legs hurt so I went home.[...] I couldn’t go because of my leg." (Client P, Da Nang)

Another client reported on the impact of their chronic headaches, “I am forgetful… I often have headaches, so I don't remember much. Sometimes when talking to my children, I cannot hear anything if they speak in a low voice.” (Client Q, Thanh Hoa) Lastly, one client reported challenges with reading the workbook due to poor eyesight:

“Q: Can you read this book yourself?

A: No.

Q: Can your daughter read it [for you]?

A: Recently, she [hasn’t].

Q: Is she busy?

A: Yes.

Q: Did your husband read it [for] you?

A: No, he is illiterate.” (Client R, Ben Tre)

Stigma was identified by both clients and providers as a barrier to participation in the SSM intervention, although it was more frequently brought up by providers. Providers shared how not all clients were receptive of engaging with the SSM and providers once it was underway and how some clients dropped out of the intervention or were hesitant to open up to them because of the negative associations around depression. As one provider reported:

“Q: When you first contact the client, are there any advantages or disadvantages [as a social collaborator]?

A: Many [households] are welcoming… I talk to them about this program [and] they try to understand and [are] supportive, but there are many houses that [don’t] understand so they get annoyed.

Q: So, they get annoyed even when you ask them questions?

A: Yes.” (Provider P, Quang Nam)

Another provider stated:

“In this [locality], when we find a client, that client is assigned a [social] collaborator. [Social collaborators] consult with the client following the PHQ-9 assessment, but that client [does not] want to accept that she has depression because she is scared that people around her [will] know about her [disease]. For example, a client… dropped out the program because she [didn’t] want to share that she has depression.” (Provider T, Ben Tre)

Another provider similarly reported:

“Q: And with regards to the difficulties and clients avoiding you: at first, they’d already agreed to meet, but why [might] they avoid seeing you?

A: From my point of view, maybe they were afraid of being interviewed. Many clients might think that I would [schedule an] interview with them to swear at them because they are crazy…” (Provider V, Da Nang)

Participants mentioned that some of these negative effects could be mitigated by building trust over time (as described previously), and “softening” the language around the initial approach:

“Q: [Is there anything] clients don’t understand when they first learn about depression? How do they react?

A: They just think that we judge them, [so] we don’t say that they [have] depression.

Q: Then how do you say it?

A: I just say, today we have some doctors coming to talk and give us advice, but I [don’t] say that they [have] depression. I wouldn’t dare to.

Q: So, you introduce them to the program first and you don’t [mention] depression?

A: I [don’t] say anything about that. I’m afraid that they would not feel good, and they wouldn’t [attend].” (Provider W, Quang Nam)

Providers also shared that one of the strategies they used to gain the trust of clients is to first gain the support of family members. While some families did not want to engage with social collaborators due to low mental health awareness, many were happy to welcome the social collaborators, encouraging their family member with depression to participate, “For example, [clients] avoid me… they do not want to see and talk with anyone at first, so I have to encourage their family, then [I will] gradually contact them. It is difficult to see [the client] immediately.” (Provider Q, Quang Ninh) Another provider shared:

“If their [condition is serious], they need help from their family members… When we guided them, we told them that there are many activities [they can try and] have fun. If they were too sad, we showed them how to [develop routines], for example: wake up [early], clean the house and do physical exercise [every day]. I would recommend 30 minutes for exercise, but if that’s not possible we can [aim for] 20 minutes. Often, they didn’t want to, so we have to create motivation… their family members have to join them to let them get used to these activities.”(Provider R, Da Nang)

Clients shared how they were fortunate to have support in their communities, especially from the amiable and supportive social providers who encouraged them to establish relationships with local associations and clubs. While it seems that stigma was a barrier for some clients, those who followed the suggestions and advice of their providers were able to build social connections with the help of their providers and reported feeling welcomed by their community.

## Discussion

Findings show that most clients reported being satisfied overall with the intervention delivered by the social collaborators. They shared their gratefulness for the program and for the care and support offered to them by the social collaborators who visited their homes and provided interpersonal connections. This is despite some of the early reluctance they expressed towards engaging with their providers, along with other contextual barriers. Participants eagerly offered insights into their daily lives with the interviewers, sharing both their struggles and aspirations.

### Barriers to participation

Through interviews with both clients and providers, depression was identified as being firmly linked to life circumstances. Risk factors for mental health conditions like depression include economic disparities, domestic and partner violence, and poverty [30, 31]. People living in LMICs such as Vietnam are exposed to a constellation of these stressors that make them vulnerable to developing psychological symptoms and/or mental disorders [32, 33]. This is reflected in what clients and providers chose to share with us about their lives.

While the inclusion criteria for participating in the RCT required clients to be literate, some shared that they were unable to read the workbook due to vision loss or deterioration related to aging. These clients often required the help of social collaborators or often younger family members to guide them through the workbook, which meant that they could not read it without assistance. Another physical challenge identified by elderly clients was chronic mobility struggles. When invited by social collaborators to join neighborhood meetings or local union gatherings to strengthen social connections, which aligns with the “reactivating your life” component of the SSM intervention, they were unable to accept due to physical struggles leaving their home.

### Situating and contextualizing depression

According to Shepherd et al., [34] “economic, social, and environmental conditions into which a person is conceived, born, reared, educated, eats, sleeps, lives, works, and receives health and social care” have a cumulative effect across a lifetime [34]. Kessler et al. (2003) found a higher 12-month incidence of major depressive disorder among those living in or near poverty [35]. Rates of depression, anxiety, and suicide have been found repeatedly to correlate negatively with income [30, 36–38] and employment [39, 30]. Several longitudinal studies have examined the relationship between depression and socioeconomic status and concluded that the causal direction runs from socioeconomic status to depression [40, 41]. This was reflected in the client experiences, where both physical and mental illnesses, which are chronic, disabling and all-consuming, stem from and lead to continued poverty. For example, a number of the clients reported financial difficulties including struggling to make ends meet by performing various jobs and tasks such as “selling soy milk” (Client B), seeeling scrap iron (Client C) and “rel[ying] on seasonal work” (Client E). Many of the clients were either widows and were required to take on the responsibility of being the primary breadwinner or had adult dependents (e.g., ill husband, adult unemployed daughter) who relied on their provisions as well as their care.

Addressing the mental health of participants cannot be done in a silo separate from acknowledging their socioeconomic status and life circumstances. As Holland (2018) wrote, “mental distress is often individualized and disconnected from social, political and economic conditions” [42]. Public-health crises such as the COVID-19 pandemic may worsen population mental health in general, but tend to disproportionately affect those living in poverty [43]. In addition, increased worries and uncertainty, and worsened physical health can impair mental health, in turn reducing employment and income [31]. Results were reflective of this as clients shared the stresses placed on them by their “financial struggles” and “worsening financial situation[s]”, which contributed to their physical and mental health concerns such as difficulty focusing (Client A), “feelings of stress and despair along with heart problems” (Client D), and feeling difficulty to “continue on” (Client E). These stressors were a significant contribution to their depression, and their depression in turn made it challenging for them to find better work.

In light of the urgent need globally to reduce the treatment gap by increasing interventions and policies that help to address some of the social and economic determinants of mental health, there has been calls globally to integrate mental health programs into other programs that target the determinants, while acknowledging the considerable budget constraints facing community-based mental health care. For example, the Lancet Commission on Global Mental Health and Sustainable Development recommended in their 2018 report an integration of mental health programs into development or aid programs for populations at an increased risk due to two or more social determinants [44]. Similarly, the Basic Needs Network advocates for the integration of their mental health programs into income generation and community development opportunities [45]. 

### Facilitators and benefits – reflections on SSM

Both clients and social collaborators shared their satisfaction with the relationships they built as a result of participating in the SSM intervention. Clients found the support of social collaborators to be invaluable while social collaborators, most of whom were already working in their communities as village health workers, also found happiness and fulfillment in their roles helping to raise mental health awareness.

As observed in other examples of task-shifting approaches from around the world and specifically in LMICs, the lay community health workers form the soul of the model, showing extraordinary empathy and compassion, filling multiple roles often out of necessity and with limited compensation. Nading (2013) followed community health workers contributing to dengue prevention campaigns in Nicaragua and observed that they provide a wide range of support that extend beyond their designated formal role, including supporting families with food security, providing necessities including clothing and school supplies, and offering comfort and support to their clients [46].

Successful social collaborators are those who have strong interpersonal skills to support building the important connection with participants, which was identified as one of the most significant factors in a successful ongoing relationship with clients. This influenced client adherence to the programs they helped promote and in turn improved client outcomes. Meas (2016) writes that community health workers are “social actors within the communities that they serve… [with] social and psychological lives that influence health policy and programs, whether intended or unintended.” More than administrators of biomedical interventions, they engage in “serious social and emotional labor” [47]. Maes further argues for the importance of advocating and working toward policies that are aimed towards creating secure employment opportunities for CHWs with appropriate compensation [47].

### Limitations and future research

As mentioned earlier in the results, 4 of the 45 client participants were male (8.9%). Although this is reasonably representative of the sample composition from the MAC-FI RCT, where 15.4% were males despite repeated attempts to try and recruit more men, this limitation should be noted again here as consequently the data may be more specific to the experience of women, who may have different experiences in terms of interactions with social collaborators (who were also mostly female) and participating in social activities. Women, however, are about twice as likely to develop depression than men [48]. Reasons for this are varied and include some of the aforementioned risk factors such as domestic violence, along with genetic and biological factors [49].

In addition, participants (clients and providers) were a convenience sample from the MAC-FI study and were asked to share their experiences retroactively. Data collection for the MAC-FI RCT occurred between July 2016 and November 2017, while interviews for the IRIS-DSV project were conducted between November 2018 and May 2019. Given the elapsed time between MAC-FI and IRIS-DSV, participants may not be entirely accurate and comprehensive in their reports. However, many of the clients and providers remained connected, though in a more minimal and less formal capacity following the RCT as they were from the same communities, which may have served as a reminder of their participation and experiences with SSM.

Amidst ongoing funding constraints and constraints on in-person care in the context of the COVID-19 pandemic, the research team, with direction from the Government of Vietnam, has shifted to delivering the SSM intervention through digital means in the form of a smartphone-based app. The team, in partnerhip with a local software development company, has adapted the in-person SSM intervention to a smartphone app (VMood), with contents of the workbook being delivered digitally and support provided remotely through the app by trained MOLISA social workers. The team is currently conducting an RCT to examine the effectiveness and cost-effectivenes of the app, informed by findings from this work on the importance of the role of lay health workers in implementing task-shifting interventions, albeit in a digital format.

## Conclusion

Findings from this study demonstrate the importance of the client-provider relationship and family and community connections in participating and engaging with a task-shifting intervention for depression. This successful relationship building was supported in large part by the interpersonal and relational skills of the providers. The relationship with providers offered clients social support and companionship and the SSM taught them evidence-based techniques to manage their depression. These factors will need to be taken into consideration for future scale-up of the SSM intervention, whether delivered in-person or digitally.

As mentioned, the team has adapted the in-person SSM intervention into a digital format, to be delivered via a smartphone-app (VMood), and is currently testing its effectiveness in an RCT in Vietnam. While evidence supports the effectiveness of digital interventions in treating a variety of health conditions [50–52], including depression [53–56], a digital version of the SSM intervention should be developed while keeping in mind that there is a significant benefit in having a sense of hope, community, and relationship with support provided by a lay health worker. The interpersonal factors that are key facilitators to the uptake and adherence of the in-person SSM may be difficult to simulate in a smartphone app; however, one of the key benefits of a digital intervention is the convenience. If clients are able to communicate with a social worker through the app at a time when convenient for them, it may facilitate access to support when required and address barriers of *clients’ responsibilities* and *clients’ health conditions* identified by participants. In addition, a smartphone app allows for privacy which protects clients from experiencing potential *consequences of* *stigma, *another barrier identified by participants. Ultimately, a model that combines delivery of the workbook through an app, along with limited support provided by lay health workers through similar digital means, may be the most viable solution.

## Supplementary Information

Supplementary Material 1

## Data Availability

The datasets used and/or analysed during the current study are available from the corresponding author on reasonable request.

## Abbreviations

WHO: World Health Organization
LMICs: Low- and Middle-Income Countries
SSM: Supported Self-Management
MAC-FI: Mental Health in Adults and Children – Frugal Innovations Study
GCC: Grand Challenges Canada
RCT: Randomized Controlled Trial
ASW: Antidepressant Skills Workbook
MOLISA: Ministry of Labour, Invalids and Social Affairs
IRIS-DSV: Implementation Research to Improve Scale-Up of Depression Services in Vietnam
PHAD: Institute of Population, Health and Development
F: Female
