# Supplementary material for: Client experiences of a task-shifting supported self-management intervention for depression in Vietnam
Source: BMC Health Serv Res. 2024 Dec 18;24:1563. doi: 10.1186/s12913-024-12036-2 (PMC11654267; doi:10.1186/s12913-024-12036-2)
Supplement: Supplementary file 1 — Supplementary Material 1 [file 12913_2024_12036_MOESM1_ESM.docx]

**Appendices**

**Appendix A: Client Interview Schedule**

***[Researcher to introduce and complete informed consent form with participant]***

1. Introduction to study:

Hello. Thank you for taking the time to participate in this interview for the Implementation Research to Improve Scale-Up of Depression Services in Vietnam (IRIS-DSV) study. The researchers on this team also conducted the Mental Health in Adults and Children – Frugal Innovations (MAC-FI) study. The MAC-FI study tested whether the Supported Self-Management intervention (from hereon in referred to as “SSM”) helped people with symptoms of depression to feel better. Now, we are conducting the IRIS-DSV study to help us understand how to make sure the SSM intervention works best for people in Vietnamese communities. You were asked to participate in the IRIS-DSV study because you participated in the MAC-FI study. This interview will help us understand several things related to the SSM program. First, it will help us to understand what you liked about the SSM, what you did not like, and what could be improved. Second, it will help us to understand any differences in interest and use of the SSM by different types of people, including men and women, and people who live in urban areas and rural areas.

We will be engaging in similar discussions with other patients and providers from the MAC-FI study in both urban and rural locations so we can better understand these differences. We will also be conducting interviews with experts in the field of mental health in Vietnam in order to understand how the SSM could be implemented on a larger scale. Overall, these interview discussions will help us to better understand how the SSM can work best for people with symptoms of depression in Vietnam and how to improve it so we can make sure it is available to everyone who needs it in the future.

During the interview, which will take approximately 30-60 minutes, I will ask you questions and invite you to share your opinions openly. There are no right or wrong answers and your experiences are important. We are here to learn from you.

As we discussed when you signed the consent form, we will record this interview so that we can capture all of your responses. [Researcher #2 Name] will also be taking notes. These notes will not contain your name or information that could be used to identify you. We will remove your name and any information that could potentially be used to identify you when we transcribe the recording and in any material we share about our findings. The notes and completed transcriptions will be stored in RADAR, SFU’s online repository, for future use in open access initiatives.

Finally, your participation in this interview is totally voluntary and you are welcome to stop at any time. There will be no negative consequences if you decide to stop before the interview is over, or if you decide you would rather not answer any of the questions.

Do you have any questions?

2. Warm up and ice-breaker:

- How long have you lived in this commune?
- What is your job or main occupation? [If patient seems shy or nervous, interviewer to ask polite probing questions about job or other occupation, e.g., student, stay-at-home parent, etc., to put patient at ease]

3. Determining patient category (e.g., participated in intervention, began intervention but did not complete, or was referred but did not participate)

- As we talked about earlier, you were invited to take part in this interview because you were previously referred to the SSM intervention. Can you please tell me whether you participated in SSM?
- If yes, did you complete the full two months of SSM?

4. Experience with screening, referral and initial uptake of SSM

***For all patients:***

Experience with screening process: With the help of a health worker, you completed a questionnaire that helped to identify that you were experiencing symptoms of depression. Based on what you remember, how did you find the experience of completing this questionnaire? [Probing questions: Did you understand what the screening was for? Did the healthcare worker explain what the results meant to you? How did you feel about this experience?]

Experience with referral process: After completing the questionnaire and discussing the results with the health worker, you were referred to take part in the SSM intervention. Based on what you remember, can you tell me about this experience? [Probing questions: Did the health worker do a good job of explaining what SSM was? Did the health worker answer any questions you had about SSM?]

***For patients who DID NOT participate in SSM:***

Uptake of SSM Why did you decide not to participate in SSM?

***For patients who DID participate in SSM:***

Uptake of SSM Why did you decide to participate in SSM?

5. Experience with SSM intervention

***For patients who DID participate [Interviewer to bring copy of Antidepressant Skills Workbook for participants’ reference and to refresh memory]:***

SSM Materials Can you tell us about your experience using the Antidepressant Skills Workbook? What did you like about it? What did you not like about it? What, if anything, could make it better?

Relationship with provider Who supported you in using SSM [Probing questions: Was it a social worker or a social collaborator? A woman or a man?] Could you tell us about your experience working with them? What did you like about it? What did you not like about it? What, if anything, could make it better?

Perceived benefits Did participating in SSM help you to feel better? If so, what changes did you notice? What aspects of SSM were particularly helpful/ unhelpful? Would you recommend SSM to a friend or family member experiencing depression?

***For patients who STOPPED participating in SSM after initial uptake***

Reasons for non-adherence Why did you decide not to continue SSM? [Probing questions: Was it the materials? The relationship with the provider? Other factors?] If you had the option now, would you decide to complete SSM?

***For patients who DID NOT participate in SSM [Interviewer to describe the SSM intervention and show the Antidepressant Skills Workbook to refresh memory]:***

Impression of SSM intervention Based on what you know about SSM, if you were referred, today would you participate? Why or why not? [Probing questions: Is the material easy to use? Does your relationship with your provider support this? Other factors?]

6. Other Comments

- Do you have any other comments or suggestions you would like to share about your experience with screening, referral, or using the SSM intervention?

**Appendix B: Provider Interview Schedule**

***[Researcher to introduce and complete informed consent form with participant]***

1. Introduction to study:

Hello. Thank you for taking the time to participate in this interview for the IRIS-DSV study. As you know, the researchers on this team also conducted the MAC-FI study. The MAC-FI study tested the effectiveness of Supported Self-Management for helping people with symptoms of depression. Now, the IRIS-DSV study will help us understand more about how to make sure the SSM intervention works best for people in Vietnamese communities. You were asked to participate in the IRIS-DSV study because you participated in the MAC-FI study. This interview will help us understand several things related to the Supported Self-Management program. First, it will help us to understand what you liked about SSM, what you didn’t like, and what could be improved. Second, it will help us to understand any differences in interest and use of SSM by different types of people, including men and women and people who live in urban areas and rural areas.

We will be conducting similar discussion with patients and other providers from the MAC-FI study in both urban and rural locations so we can understand these differences. We will also be conducting interviews with experts in the field of mental health in Vietnam in order to understand how the SSM could be implemented on a larger scale. Overall, these interview discussions will help us to better understand how SSM can work best for people with symptoms of depression in Vietnam and how to improve it so we can make sure it is available to everyone who needs it in the future.

During the interview, which will take approximately 30-60 minutes, I will ask you questions and invite you to share your opinions openly. There are no right or wrong answers and your experiences are important. We are here to learn from you.

As we discussed when you signed the consent form, we will record this interview so that we can capture all of your responses. [Researcher #2 Name] will also be taking notes. These notes will not contain your name or information that could be used to identify you. We will remove your name and any information that could potentially be used to identify you when we transcribe the recording and in any material we share about our findings. The notes and completed transcriptions will be stored in RADAR, SFU’s online repository, for future use in open access initiatives.

Finally, your participation in this interview is totally voluntary and you are welcome to stop at any time. There will be no negative consequences if you decide to stop before the interview is over, or if you decide you would rather not answer any of the questions.

Do you have any questions?

2. Warm up and ice-breaker:

- Could you tell me a bit about your job as a social worker/ social collaborator? [Probing questions: How long have you been doing this job? Could you tell me about your main tasks?]
- Approximately how many patients have you worked with to deliver SSM?

3. SSM Intervention Delivery

*We’ve brought a copy of the workbook with us in order to refresh your memory. You can refer to this throughout the interview.*

***[Interviewer to give copy of Antidepressant Skills Workbook to participant]***

Referral of patients to SSM Please describe the process of patients being referred to you for SSM by a health worker. What works well about this process? What, if any, challenges exist?

Introducing SSM Please describe how you introduce SSM to patients. How do you describe the program to them? What works well about this process? What, if any, challenges have you encountered?

Using SSM workbook Please describe the process of using the Antidepressant Skills Workbook (ASW) with patients. Do you give the workbook to every patient you work with? (If no, why not?) How do patients usually respond to being given the workbook? If they are hesitant, do you encourage them to use it? (If yes, how? If no, why not?)

Patients guided through SSM Please describe the process of guiding patients through the SSM intervention. In general, how often do you meet with the patient? How do you provide support to patients? What has worked well in this process? What if any challenges have you encountered?

4. Need for adaption

ASW workbook Based on your experience in supporting patients to use the ASW, what works well about the book? What, if anything, would you change?

Provider-patient relationship Based on your experience guiding patients through SSM, what works well about this process? What, if anything, would you change?

Other Based on your experience with SSM, are there any other aspects that work well or that you think should be changed?

5. Other Comments

- Do you have any other comments or suggestions you would like to share about your experience delivering the SSM intervention?

**Appendix C: Client Recruitment Script**

*[This will be used for initial contact with potential participants, either by telephone or email]*

Dear [*name*],

We are contacting you to invite you to participate in a research study that is being carried out in partnership between the Institute of Population, Health and Development and Simon Fraser University (Vancouver, Canada).

You may have participated in a previous study (Mental Health in Adults and Children – Frugal Innovations [MAC-FI]), where you were offered a community-based mental health intervention called ‘Supported Self-Management (SSM)’. The goal of the study was to examine whether the SSM was successful for helping to lower symptoms of depression and to help improve the ability of people with symptoms of depression to take part in their daily activities.

The purpose of this research is to understand the SSM program, including how to make it accessible to all people in Vietnam who experience depression. As a patient, you will be asked to participate in an in-person interview that will take approximately 30-60 minutes. The session will be audio recorded and notes will be taken. Depending on the extent to which you participated in the SSM, you will be asked about your experiences, such as why you decided to participate or not participate, and what made you decide to either continue or not continue with SSM. If you did participate, we will also ask you about what you found helpful/did not find helpful with the SSM program. Ultimately, this will help us understand how the SSM can offer care to people with depression in Vietnam and ensure they receive better and more appropriate care.

Participation is entirely voluntary and you may withdraw at any time. Your responses will be kept confidential. You will receive a small gift of a 100,000 Vietnamese Dong phone credit.

If you are interested in this study, please contact [*name and contact information of coordinator at PHAD who will be involved with study; TBD*]

**Appendix D: Provider Recruitment Script**

*[This will be used for initial contact with potential participants, either by telephone or email]*

Dear [*name*],

We are contacting you to invite you to participate in a research study that is being carried out in partnership between the Institute of Population, Health and Development and Simon Fraser University (Vancouver, Canada).

In a previous study you participated in (Mental Health in Adults and Children – Frugal Innovations [MAC-FI]), the researchers trained commune health workers and social workers, such as yourself, to screen patients for depression and to offer a community-based mental health intervention called ‘Supported Self-Management (SSM)’. The goal of the study was to examine whether the SSM was successful for helping to lower symptoms of depression and to help improve the ability of people with symptoms of depression to take part in their daily activities.

The purpose of this research is to understand the SSM program, including how to make it accessible to all people in Vietnam who experience depression. As a health care worker, you will be asked to participate in an interview that will take approximately 30-60 minutes. The session will be audio recorded and notes will be taken. You will be asked questions related to your experience using the SSM intervention, including the successes and any challenges you experienced. Ultimately, this research will help us understand how the SSM can offer care to people with depression in Vietnam and ensure they receive better and more appropriate care.

Participation is entirely voluntary and you may withdraw at any time. Your responses will be kept confidential. You will receive a small gift of a 100,000 Vietnamese Dong phone credit.

If you are interested in this study, please contact [*name and contact information of coordinator at PHAD who will be involved with study; TBD*]
